# Detection of neuron‐derived cfDNA in blood plasma: A novel screening and monitoring approach for Alzheimer's disease and other neurodegenerative conditions

**DOI:** 10.1002/alz70856_103924

**Published:** 2025-12-26

**Authors:** Chad Pollard

**Affiliations:** ^1^ Brigham Young University, Provo, UT, USA; Resonant, Heber City, UT, USA

## Abstract

**Background:**

Current diagnostic standards for Alzheimer's disease (AD), amyloid and tau biomarkers, provide valuable insights but are limited as early screening tools and lack applicability across neurodegenerative conditions. Our preliminary findings show cortical neuron‐derived cell‐free DNA (cfDNA) in blood correlates with mild cognitive impairment (MCI) and AD, offering a neuron‐specific measure of neurodegeneration^1^. However, this cfDNA detection relied on bisulfite sequencing at a single cortical neuron‐specific site, constrained by DNA damage, PCR biases, and limited scope. We present a nanopore sequencing approach that eliminates these limitations, enabling analysis of thousands of cell type‐specific loci with superior sensitivity and accuracy. This approach is also applied to dopaminergic neurons in Parkinson's disease (PD) and spinal motor neurons in amyotrophic lateral sclerosis (ALS), positioning cfDNA as a promising target for early detection, staging, and management of neurodegenerative diseases.

**Methods:**

DNA was extracted from blood plasma and cortical, spinal motor, and dopaminergic neurons. Whole‐genome sequencing was performed using nanopore technology (PromethION, Oxford Nanopore Technologies) to enable native DNA methylation analysis without bisulfite conversion or amplification. Neuron‐specific methylation signatures were identified across the genome, targeting differentially methylated regions (DMRs) unique to each cell type. cfDNA from blood samples of controls and patients with MCI/AD (*n* = 80), PD (*n* = 64), and ALS (*n* = 70) was sequenced and analyzed at neuron‐specific loci.

**Results:**

Mixed DNA dilutions demonstrated >96% accuracy in assigning cortical, dopaminergic, and spinal motor neurons, targeting 4,152,224 loci for cortical neurons, 130,137 loci for dopaminergic neurons, and 1,427,252 loci for spinal motor neurons. cfDNA levels were significantly elevated in MCI/AD (*p* = 2.2e‐6), PD (*p* = 1.014e‐14), and ALS (*p* = 2.056e‐14).

**Conclusion:**

Nanopore sequencing achieves accurate neuron‐specific cfDNA methylation analysis and demonstrates potential as a minimally invasive target for early detection, staging, and monitoring of neurodegenerative diseases. A longitudinal AD validation study (*n* = 775) is underway in collaboration with the University of Kansas Alzheimer's Disease Research Center to assess early screening and disease monitoring applications in AD.

**References**

1. Pollard, et al. Detection of neuron‐derived cfDNA in blood plasma: a new diagnostic approach for neurodegenerative conditions. *Frontiers in Neurology*. 2023.

